# *RBOH1*-dependent H_2_O_2_ production and subsequent activation of MPK1/2 play an important role in acclimation-induced cross-tolerance in tomato

**DOI:** 10.1093/jxb/ert404

**Published:** 2013-12-09

**Authors:** Jie Zhou, Xiao-Jian Xia, Yan-Hong Zhou, Kai Shi, Zhixiang Chen, Jing-Quan Yu

**Affiliations:** ^1^Department of Horticulture, Zijingang Campus, Zhejiang University, Yuhangtang Road 866, Hangzhou 310058, PR China; ^2^Key Laboratory of Horticultural Plants Growth, Development and Quality Improvement, Agricultural Ministry of China, Yuhangtang Road 866, Hangzhou 310058, PR China; ^3^Department of Botany and Plant Pathology, Purdue University, West Lafayette, IN 47907-2054, USA

**Keywords:** Cross-tolerance, hydrogen peroxide, mitogen-activated protein kinase, reactive oxygen species, *Respiratory burst oxidase homologue 1*, signal transduction, *Solanum lycopersicum*.

## Abstract

H_2_O_2_ and mitogen-activated protein kinase (MAPK) cascades play important functions in plant stress responses, but their roles in acclimation response remain unclear. This study examined the functions of H_2_O_2_ and MPK1/2 in acclimation-induced cross-tolerance in tomato plants. Mild cold, paraquat, and drought as acclimation stimuli enhanced tolerance to more severe subsequent chilling, photooxidative, and drought stresses. Acclimation-induced cross-tolerance was associated with increased transcript levels of *RBOH1* and stress- and defence-related genes, elevated apoplastic H_2_O_2_ accumulation, increased activity of NADPH oxidase and antioxidant enzymes, reduced glutathione redox state, and activation of MPK1/2 in tomato. Virus-induced gene silencing of *RBOH1*, *MPK1*, and *MPK2* or *MPK1/2* all compromised acclimation-induced cross-tolerance and associated stress responses. Taken together, these results strongly suggest that acclimation-induced cross-tolerance is largely attributed to *RBOH1*-dependent H_2_O_2_ production at the apoplast, which may subsequently activate MPK1/2 to induce stress responses.

## Introduction

Plants are often exposed to various unfavourable environmental stresses (i.e. extreme temperatures, drought, salt, fungi, bacteria, and herbicides) throughout their life cycles. To survive against stresses, plants have intricate defence mechanisms to increase their tolerance. Acclimation is the process in which an individual organism adjusts to a gradual change in its environment (such as a change in temperature, humidity, or photoperiod), allowing it to maintain performance across a range of environmental conditions. Recent studies have also revealed that there exists a kind of adaptation mechanism called cross-tolerance, whereby plants tolerant to one stress are often tolerant to a range of other stresses (Pastori and Foyer, 2002; Capiati *et al.*, 2006; Suzuki *et al.*, 2012). Increased anoxia tolerance was reported in *Arabidopsis* plants in response to heat (Banti *et al.*, 2008), while NaCl and wounding induced resistance to UV-B in barley and salt tolerance in tomato plants (Capiati *et al.*, 2006; Carkirlar *et al.*, 2008). While the capacity to acclimate to novel environments has been well documented in different plant species, very little is still known about the in-depth mechanisms of such acclimation in plants.

Cold acclimation has been most studied in terms of the physiological and molecular mechanisms in plants. At the metabolic level, acclimation induced accumulation of osmolytes, cryoprotectants, and abscisic acid (ABA) and production of compatible solutes (e.g. proline, raffinose, and glycine betaine) to stresses such as low non-freezing temperatures and moderate light (Browse and Xin, 2001). Acclimation is also able to stabilize proteins and cellular structures and to maintain cell turgor by osmotic adjustment and cellular redox balance (Janska *et al.*, 2010). At the molecular level, secondary messengers such as cytosolic Ca^2+^, nitric oxide (NO), ABA, and reactive oxygen species (ROS) such as hydrogen peroxide (H_2_O_2_) are found to be involved in the perception and transduction of low temperature signal to trigger cold acclimation-induced changes in physiological processes (Zhao *et al.*, 2009; Janska *et al.*, 2010; Zhou *et al.*, 2012). Foliar application of these chemicals increased the tolerance to an array of stresses such as drought, salt, and extreme temperature. For example, H_2_O_2_ enhanced the transcription of a subset of stress-responsive genes and the antioxidant capacity of cells by increasing the activities of antioxidant enzymes, such as superoxide dismutase (SOD), ascorbate peroxidase (APX), catalase (CAT), and glutathione reductase (GR), and the biosynthesis of non-enzymic antioxidants such as ascorbic acid and glutathione with an increase in the ratio of reduced glutathione (GSH) to oxidized glutathione (GSSG) (Thannickal and Fanburg, 2000; Jiang *et al.*, 2012). Maintaining the redox homeostasis is a prerequisite for the development of tolerance against both biotic and abiotic stresses (Foyer *et al.*, 1997; Mou *et al.*, 2003; Jiang *et al.*, 2012). ROS, especially H_2_O_2_ generated by NADPH oxidases encoded by *Respiratory Burst Oxidase Homologue* (*RBOH*) genes play important roles in plant responses to biotic and abiotic stresses (Torres *et al.*, 2002; Kwak *et al.*, 2003; Yoshioka *et al.*, 2003; Torres and Dangl, 2005; Marino *et al.*, 2012). In *Arabidopsis*, there are increased transcript levels of *rbohD* and *rbohE* and ROS accumulation in response to infection with virulent *Pseudomonas syringae* pv. tomato DC3000, and these responses were greatly compromised in *rbohD* and *rbohE* mutants (Torres *et al.*, 2002; Kwak *et al.*, 2003). Similarly, silencing *RBOHA* and *RBOHB* in *Nicotiana benthamiana* plants reduced ROS production and compromised resistance to *Phytophthora infestans* (Yoshioka *et al.*, 2003). Meanwhile, ROS, NO, cytosolic Ca^2+^, and plant hormones such as ABA and brassinosteroids (BRs) crosstalk in stress responses (Dempsey and Klessig, 1995; Desikan *et al.*, 2004; Wendehenne *et al.*, 2004; Xia *et al.*, 2009; Cui *et al.*, 2011). For example, H_2_O_2_ cooperates with NO in plant HR/cell death and abiotic stresses (Wendehenne *et al.*, 2004; Cui *et al.*, 2011) and plays a critical role in BR-induced stress tolerance (Xia *et al.*, 2009). Expression of *RBOHs* is also regulated by plant hormones such as ABA and BRs. Elevation of ABA and BR levels resulted in increased production of H_2_O_2_ via RBOHs together with increased tolerance against a subset of stresses (Xia *et al.*, 2009; Zhang *et al.*, 2009). A major contributor to induced ROS production for RBOHs may act as converging regulators in the orchestration of plant adaptation to environmental stresses (Marino *et al.*, 2012). However, there has been no genetic evidence to show that RBOHs are involved in acclimation-induced cross-tolerance.

The mitogen-activated protein kinase (MAPK) cascade, minimally composed of a MAPK kinase kinase, MAPK kinase, and a MAPK, is one of the major pathways by which extracellular stimuli are transduced into intracellular signals in plant stress responses (Tena *et al.*, 2001; Zhang and Klessig, 2001). For example, wounding induced increased activation of MAPKs and systemic response to insect attack in tomato leaves (Stratmann and Ryan, 1997). For its involvement in plant signal transduction in response to biotic and abiotic stresses, MAPK signalling also interacts with ROS, NO, and ABA signalling pathways (Lu *et al.*, 2002; Samuel and Ellis, 2002; Mittler *et al.*, 2004; Pitzschke *et al.*, 2009). BR- and ABA-induced apoplastic H_2_O_2_ could activate MAPKs in plants, leading to enhanced antioxidant defence system in leaves of tomato and maize (Lin *et al.*, 2009; Nie *et al.*, 2013). On the other hand, NO and NADPH oxidase-dependent oxidative bursts are also regulated by MAPK signals in *N. benthamiana* and tomato plants (Yoshioka *et al.*, 2003; Asai *et al.*, 2008; Nie *et al.*, 2013). It is, therefore, quite plausible that the MAPK cascade is also involved in acclimation-induced cross-responses to multiple stresses.

Previously, Zhou *et al.* (2012) reported the potential role of H_2_O_2_ elevation in cold acclimation in tomato plants. To obtain insights into the signalling events in acclimation-induced abiotic stress cross-tolerance in tomato, the current study investigated whether pretreatment of one type of mild stress induces acclimation to multiple types of subsequent, more severe, stress treatments and if so, how the cross-acclimation is related to apoplastic H_2_O_2_ accumulation and expression of *RBOH1*, the tomato homologue of *AtRbohF* and *NtRbohA*, which play a critical role in both stress and adaptation responses in *Arabidopsis* and tobacco, respectively (Kwak *et al.*, 2003; Yoshioka *et al.*, 2003). This work also examined how cross-acclimation and the associated stress response were affected by silencing of *RBOH1* and *MPK1* and *2*. The results have provided strong evidence that apoplastic H_2_O_2_ and *RBOH1*-mediated MPK1/2 activation plays a critical role in induction of cross-acclimation to abiotic stresses in tomato plants.

## Materials and methods

### Plant materials, virus-induced gene silencing constructs, and *Agrobacterium*-mediated virus infection

Tomato (*Solanum lycopersicum* L. cv. Condine Red) seeds were germinated in a growth medium filled with a mixture of peat and vermiculite (7:3, v/v) in trays in a growth chamber. When the first true leaf was fully expanded, seedlings were transplanted into plastic pots (15cm diameter and 15cm depth, one seedling per pot) containing the same medium and were watered daily with Hoagland’s nutrient solution. The growth conditions were as follows: a 14/10 light/dark cycle, 25/20 °C, and photosynthetic photon flux density (PPFD) 600 μmol m^–2^ s^–1^.

The tobacco rattle virus (pTRV) virus-induced gene silencing (VIGS) constructs used for silencing of tomato *RBOH1* genes was generated by cloning a 311-bp *RBOH1* cDNA fragment, which was amplified using the forward (5′-ATACGCGAGCTCAAGAATGGGGTTGATATTGT-3′) and reverse (5′-ATACCGCTCGAGCTCTGACTTATTCCTTAC-3′) primers according to Liu *et al.* (2002). The amplified fragment was digested with *Sac*I and *Xho*I and ligated into the same sites of pTRV2. The resulting plasmid was transformed into *Agrobacterium tumefaciens* GV3101. The pTRV-*MPK1*, pTRV-*MPK2*, and cosilencing pTRV-*MPK1/2* VIGS constructs were generated as described previously (Kandoth *et al.*, 2007). *Agrobacterium*-mediated virus infection was performed as previously described (Ekengren *et al.*, 2003). Plants were then kept at 23/21 °C under 125 μmol m^–2^ s^–1^ PPFD for 30 d before they were used. Leaflets in the middle of the fifth fully expanded leaves, which showed 20–30% transcript levels of control plants, were used. The expression of *MPK1* and *MPK2* in pTRV-*MPK1*, pTRV-*MPK2*, and pTRV-*MPK1/2* plants is shown in Supplementary Fig. S1A (available at *JXB* online). Each replicate had 12 plants.

### Acclimations and tolerance analysis

To investigate acclimation-induced cross-tolerance to abiotic stresses, tomato seedlings at the five-leaf stage were transferred to a growth chamber for pretreatment with cold (8 °C, 400 μmol m^–2^ s^–1^ PPFD, 3 d), paraquat (PQ; 10 μM, 25/20 °C, 400 μmol m^–2^ s^–1^ PPFD, 2 d), or drought (in growth medium with 20% moisture, 3 d). Control unacclimated plants were maintained in a growth chamber at 25/20 °C and 400 μmol m^–2^ s^–1^ PPFD. Then, acclimated and unacclimated plants were exposed to 4 °C and 400 μmol m^–2^ s^–1^ PPFD for 3 d for chilling treatment or 50 μM paraquat for 1 d for PQ treatment or were grown in drought medium (moisture less than 15%) for 3 d for drought treatment. There were four replicates and each replicate had 12 plants.

Stress tolerance was determined by analysing gas exchange and chlorophyll fluorescence. The light-saturated CO_2_ assimilation rate was determined with an infrared gas analyser-based portable photosynthesis system (LI-6400, LI-COR, Lincoln, NE, USA). The air temperature, relative humidity, CO_2_ concentration, and PPFD used for measurement of Asat were 25 °C, 85%, 380 μmol mol^–1^, and 1000 μmol m^–2^ s^–1^, respectively.

Chlorophyll (Chl) fluorescence was measured using an Imaging-PAM Chlorophyll Fluorometer equipped with a computer-operated PAM-control unit (IMAG-MAXI, Heinz Walz, Effeltrich, Germany). Seedlings were kept in the dark for approximately 30min before measurements were taken. The intensities of the actinic light and saturating light settings were 280 μmol mol^–2^ s^–1^ and 2500 μmol mol^–2^ s^–1^ photosynthetically active radiation, respectively. The maximum quantum yield of PSII (*F*_v_/*F*_m_) were measured and calculated as previously described (Zhou *et al.*, 2012).

### Analysis of H_2_O_2_

Histochemical staining of H_2_O_2_ was performed as previously described (Thordal-Christensen *et al.*, 1997), with minor modifications as described previously (Xia *et al.*, 2009). Leaf discs were vacuum infiltrated with 1mg ml^–1^ 3,3′- diaminobenzidine (DAB) in 50mM TRIS-acetate buffer (pH 3.8) and incubated at 25 °C in the dark for 6h. Leaf discs were rinsed in 80% (v/v) ethanol for 10min at 70 °C and mounted in lactic acid/phenol/water (1:1:1, v/v/v), and H_2_O_2_ accumulation was detected by an Olympus motorized system microscope (BX61, Olympus, Tokyo, Japan). H_2_O_2_ was visualized at the subcellular level using CeCl_3_ for localization (Zhou *et al.*, 2012). Sections were examined using a transmission electron microscope (H7650, Hitachi, Tokyo, Japan) at an accelerating voltage of 75kV. Electron-dense CeCl_3_ deposits which are formed in the presence of H_2_O_2_ are visible by transmission electron microscopy (Bestwick *et al.*, 1997). H_2_O_2_ in leaf tissue was extracted and analysed as previously described (Willekens *et al.*, 1997).

### Antioxidant assays

For antioxidant enzyme assays, leaf tissues (0.3g) were ground with a 2ml ice-cold buffer containing 50mM PBS (pH 7.8), 0.2mM EDTA, 2mM AsA, and 2% (w/v) polyvinylpolypyrrolidone. Homogenates were centrifuged at 12 000 *g* for 20min, and the resulting supernatants were used for the determination of enzyme activity. All steps were performed at 4 °C. An aliquot of the extract was used to determine the protein content, following the method as previously described (Bradford, 1976), using bovine serum albumin as a standard. The activity of CAT, APX, SOD, and GR were measured following the protocol used as previously described (Xia *et al.*, 2009). All spectrophotometric analyses were conducted on a SHIMADZU UV-2410PC spectrophotometer (Shimadzu, Kyoto, Japan). For the measurement of GSH and GSSG, plant leaf tissue (0.3g) was homogenized in 2ml of 2% metaphosphoric acid containing 2mM EDTA and centrifuged at 4 °C for 10min at 14 000 *g*. Total and oxidized glutathione (GSH+GSSG and GSSG, respectively) levels were determined as previously described (Griffith, 1980).

### Isolation of plasma membranes and determination of NADPH oxidase activity

Leaf plasma membranes were isolated using a two-phase aqueous polymer partition system (Xia *et al.*, 2009). The NADPH-dependent 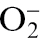-generating activity in isolated plasma membrane vesicles was determined by following the protocol used as previously described (Zhou *et al.*, 2012).

### Total RNA extraction and gene expression analysis

Total RNA was isolated from tomato leaves using Trizol reagent (Sangon, China), according to the manufacturer’s recommendations. Genomic DNA was removed using a RNeasy Mini Kit (Qiagen, Germany). Total RNA (1 μg) was reverse transcribed using a ReverTra Ace qPCR RT Kit (Toyobo, Osaka, Japan), following the manufacturer’s instructions. Gene-specific quantitative real-time PCR (qRT-PCR) primers were designed based on their cDNA sequences and are listed in Supplemental Table S1. These 10 genes encode MAPKs, defence-regulatory or -related proteinsm and antioxidant enzymes: *MPK1* (encoding mitogen-activated protein kinase 1), *MPK2* (encoding mitogen-activated protein kinase 2), *NPR1* (encoding non-expresser of PR 1), *NPR1.1* (encoding non-expresser of PR 1.1), *PR1* (encoding pathogenesis-related 1), *Fe-SOD* (encoding Fe-SOD), *Cu/Zn-SOD* (encoding Cu/Zn-SOD), *cAPX* (encoding cytosolic ascorbate peroxidase), *CAT1* (encoding catalase 1), and *GR1* (encoding glutathione reductase 1). qRT-PCR was performed using the iCycleri Q real-time PCR detection system (Bio-Rad, Hercules, CA, USA). Each reaction (25 μl) consisted of 12.5 μL SYBR Green PCR Master Mix (Takara, Chiga, Japan), 1 μl diluted cDNA, and 0.1 μmol forward and reserve primers. The cycling conditions were as follows: 95 °C for 3min, and 40 cycles of 95 °C for 10 s and 58 °C for 45 s. Tomato *Actin* was used as an internal control. Relative gene expression was calculated as previously described (Livak and Schmittgen, 2001).

### MPK1 and 2 activation assay

Tomato leaves were collected after cross-acclimation, ground in liquid nitrogen, and homogenized in an extraction buffer (100mM HEPES pH 7.5, 5mM EDTA, 5mM EGTA, 10mM DTT, 10mM Na_3_VO_4_, 10mM NaF, 50mM β-glycerophosphate, 1mM phenylmethylsulphonyl fluoride, 5 μg/ml antipain, 5 μg/ml aprotinin, 5 μg/ml leupeptin, 10% glycerol, and 7.5% polyvinylpolypyrrolidone). Homogenates were clarified by centrifugation at 16 000 *g*, extracted proteins were separated by SDS-PAGE, and MPK1 and MPK2 activation was detected by protein blotting using phospho-p44/42 MAPK (ERK1/2, Thr202/Try204) monoclonal antibody (Cell Signalling Technology, Danvers, MA, USA; Faulkner *et al.*, 2013; Nie *et al.*, 2013). ERK1/2 could specifically detect activated MPK1/2 with a molecular mass of 46 kD in tomato plants (Nie *et al.*, 2013).

### Statistical analysis

A completely randomized block design with four blocks was applied in each experiment with 10 plants as a replicate. Measurements were replicated four times and randomly arranged in each block. An analysis of variance was carried out according to the general linear model procedure of statistical analysis system (SAS). Differences between treatment means were separated by the Tukey’s test at *P* < 0.05.

## Results

### Acclimation-induced cross-tolerance is associated with increased H_2_O_2_ accumulation at the apoplast

**Fig. 1.**
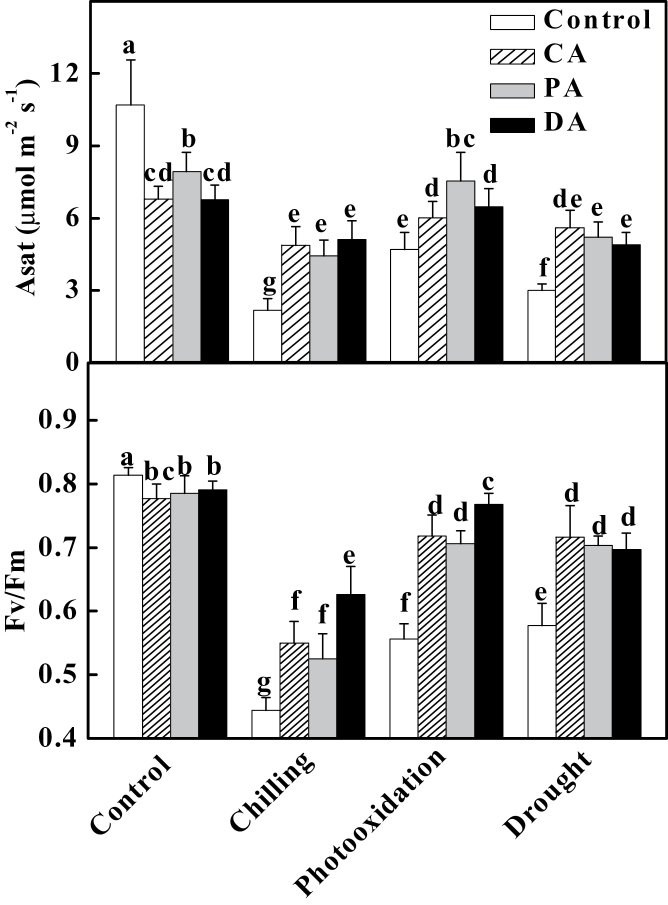
Effects of cold acclimation (8 °C, 3 d), paraquat acclimation (10 μM, 2 d), and drought acclimation (growth medium with 20% moisture, 3 d) on the light-saturated CO_2_ assimilation rate (Asat) and the maximum quantum yield of PSII (*F*_v_/*F*_m_) after chilling (4 °C, 3 d), photooxidation (50 μM paraquat, 1 d), or drought (<15% moisture, 3 d). Leaflets in the middle fifth leaf were used. Data are mean±SD of four biological replicates. Different letters above the bars indicate values that are significantly different (*P* < 0.05) according to Tukey’s test. Control, no acclimation; CA, cold acclimation; PA, paraquat acclimation; DA, drought acclimation.

To determine whether cold (CA), drought (DA), and paraquat (PA) acclimation could induce cross-acclimation in tomato, tomato plants were first unacclimated or acclimated to cold at 8 °C, 10 μM PQ, or drought soil moisture at 20% and then exposed to chilling (4 °C) for 3 d, high concentration of PQ (50 μM) for 1 d, and extreme drought (moisture less than 15%) for 3 d. Light-saturated Asat decreased 19–28% while the maximal quantum efficiency of PSII (*F*_v_/*F*_m_) decreased slightly after these acclimations (Fig. 1). However, Asat of unacclimated plants were decreased by 80, 56, and 72% while *F*_v_/*F*_m_ were reduced 45, 32, and 29% after exposure to chilling, PQ, and extreme drought stresses, respectively (Fig. 1). Importantly, CA, PA, and DA all significantly alleviated the stress-induced decreases in Asat and *F*_v_/*F*_m_, as indicated from their increases of 28–136% for Asat and 18–29% for *F*_v_/*F*_m_ as compared to unacclimated plants (Fig. 1). All these results suggested that these acclimations could induce cross-tolerance in tomato plants.

**Fig. 2.**
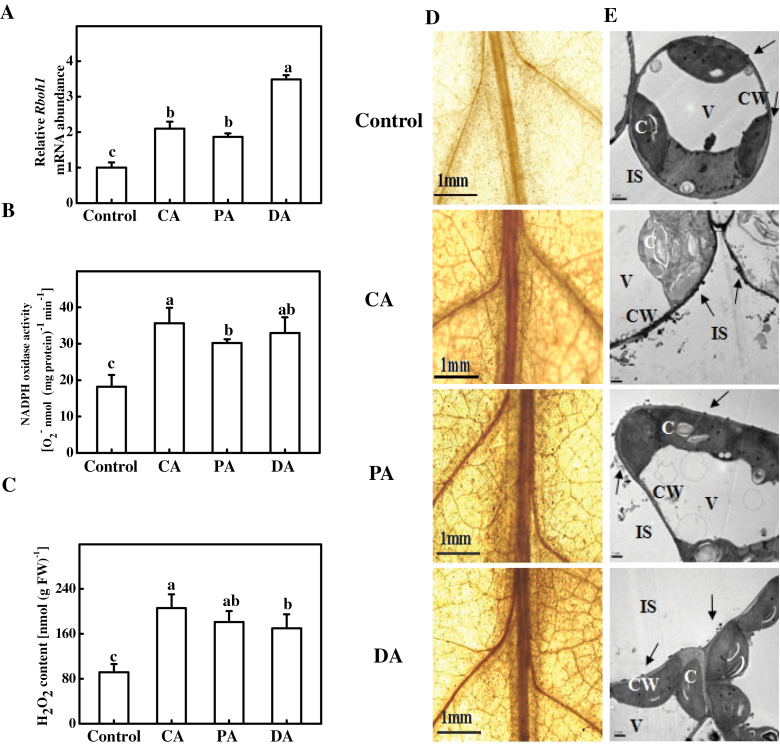
Cross-acclimation-induced changes in *RBOH1* transcript levels (A), NADPH oxidase activity (B), and H_2_O_2_ accumulation (C) in tomato leaves after acclimation to cold (8 °C, 3 d), paraquat (10 μM, 2 d), or drought (growth medium with 20% moisture, 3 d). (D) *In situ* detection of H_2_O_2_: leaf segments were loaded with DAB and incubated for 6h and H_2_O_2_ accumulation was detected by microscope; bars, 1.0mm. (E) Cytochemical localization of H_2_O_2_ in mesophyll cells of leaves, detected with CeCl_3_ staining and transmission electron microscopy: arrows, CeCl_3_ precipitates; C, chloroplast; CW, cell wall; IS, intercellular space; V, vacuole. Leaflets in the middle fifth leaf were used. Data are mean±SD of four biological replicates. Different letters above the bars indicate values that are significantly different (*P* < 0.05) according to Tukey’s test. Control, no acclimation; CA, cold acclimation; PA, paraquat acclimation; DA, drought acclimation.

A previous study (Zhou *et al.*, 2012) found that cold acclimation induced H_2_O_2_ accumulation in tomato leaves. To determine whether acclimations other than cold acclimation could also induce H_2_O_2_ accumulation at the apoplast by triggering NADPH oxidase activity, this study analysed *RBOH1* expression, NADPH oxidase activity, and H_2_O_2_ accumulation in CA, PA, and DA plants. As shown in Fig. 2, the three types of acclimation all resulted in significant increases in H_2_O_2_ accumulation, and the increase was accompanied by significant increases in both *RBOH1* expression and plasma membrane NADPH oxidase activity. *RBOH1* expression increased by 1.1-fold, 0.9-fold, and 2.5-fold after CA, PA, and DA, respectively. Similarly, NADPH oxidase activity increased by 96, 66, and 81%, and H_2_O_2_ concentration increased by 1.3-fold, 98%, and 86% after CA, PA, and DA, respectively (Fig. 2A–C).

The *in situ* detection of H_2_O_2_ using DAB staining revealed increased H_2_O_2_ accumulation in acclimated leaves, and this was especially apparent in CA plants (Fig. 2D). Using a CeCl_3_-based procedure, it was found that all these acclimations induced H_2_O_2_ accumulation in the cell walls of mesophyll cells that face intercellular spaces (Fig. 2E). These results suggest a potential role of NADPH oxidases in these acclimated plants.

### The role of *RBOH1* in acclimation-induced cross-tolerance

**Fig. 3.**
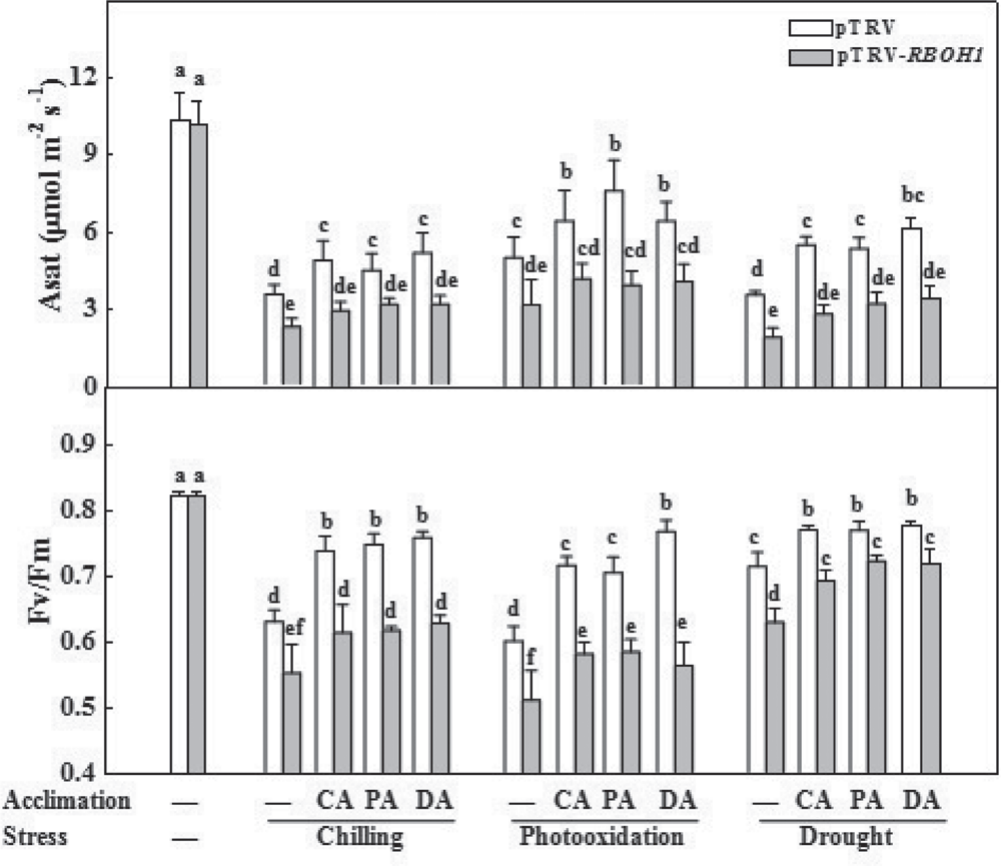
Changes in light-saturated CO_2_ assimilation rate (Asat) and maximum quantum yield of PSII (*F*_v_/*F*_m_) in response to chilling (4 °C, 3 d), paraquat (50 μM, 1 d), and drought (<15% moisture, 3d) in *RBOH1*-silenced plants after cross-acclimation pretreatment. Leaflets in the middle fifth leaf were used. Data are mean±SD of four biological replicates. Different letters above the bars indicate values that are significantly different (*P* < 0.05) according to Tukey’s test. —, no acclimation; CA, cold acclimation; PA, paraquat acclimation; DA, drought acclimation.

To determine whether acclimation-induced H_2_O_2_ accumulation at the apoplast was associated with cross-tolerance, this study compared tolerance against chilling, PQ, and drought stresses in pTRV plants and in *RBOH1*-silenced plants (pTRV-*RBOH1*). pTRV-*RBOH1* plants showed similar Asat and *F*_v_/*F*_m_ values to those of pTRV plants when they were grown under clement environments (Fig. 3). Chilling, PQ, and drought stresses all resulted in significant decreases in Asat and *F*_v_/*F*_m_ in pTRV plants and pTRV-*RBOH1* plants. Meanwhile, pTRV-*RBOH1* plants wilted earlier than pTRV plants after exposure to chilling and drought stress and showed more severe leaf bleaching after PQ stress. Importantly, decreases in Asat and *F*_v_/*F*_m_ were largely alleviated by CA, PA, and DA in pTRV plants but not in pTRV-*RBOH1* plants. Accordingly, *RBOH1* functioned as an important role in acclimation-induced cross-tolerance in tomato plants.

**Fig. 4.**
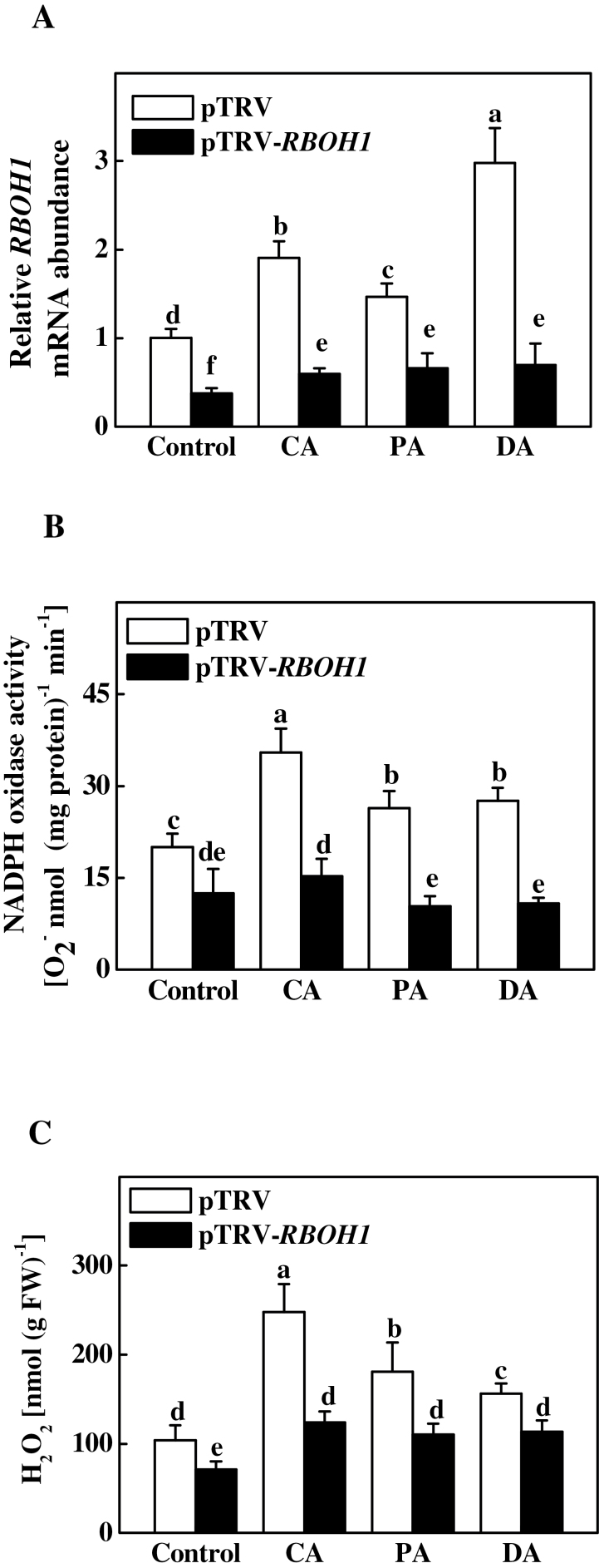
Changes in *RBOH1* transcript levels (A), NADPH oxidase activity (B), and H_2_O_2_ accumulation (C) in *RBOH1*-silenced tomato plants after acclimation to cold (8 °C, 3 d), paraquat (10 μM, 2 d), or drought (20% moisture, 3 d). Leaflets in the middle fifth leaf were used. Data are mean±SD of four biological replicates. Different letters above the bars indicate values that are significantly different (*P* < 0.05) according to Tukey’s test. Control, no acclimation; CA, cold acclimation; PA, paraquat acclimation; DA, drought acclimation; FW, fresh weight.

*RBOH1* transcript levels, NADPH oxidase activity, and H_2_O_2_ accumulation were significantly lower in pTRV-*RBOH1* plants as compared to pTRV plants under normal conditions (Fig 4, Supplementary Fig. S2). Similarly to that observed in normal plants, CA, PA, and DA resulted in significant increases in *RBOH1* transcript levels, NADPH oxidase activity, and H_2_O_2_ accumulation in the leaves of pTRV plants. Such increases in *RBOH1* transcript levels, NADPH oxidase activity, and H_2_O_2_ accumulation, however, were not observed in pTRV-*RBOH1* plants after CA, PA, and DA (Fig. 4, Supplementary Fig. S2), suggesting that *RBOH1* is necessary for CA-, PA-, and DA-induced increases in NADPH oxidase activity and H_2_O_2_ content.

**Fig. 5.**
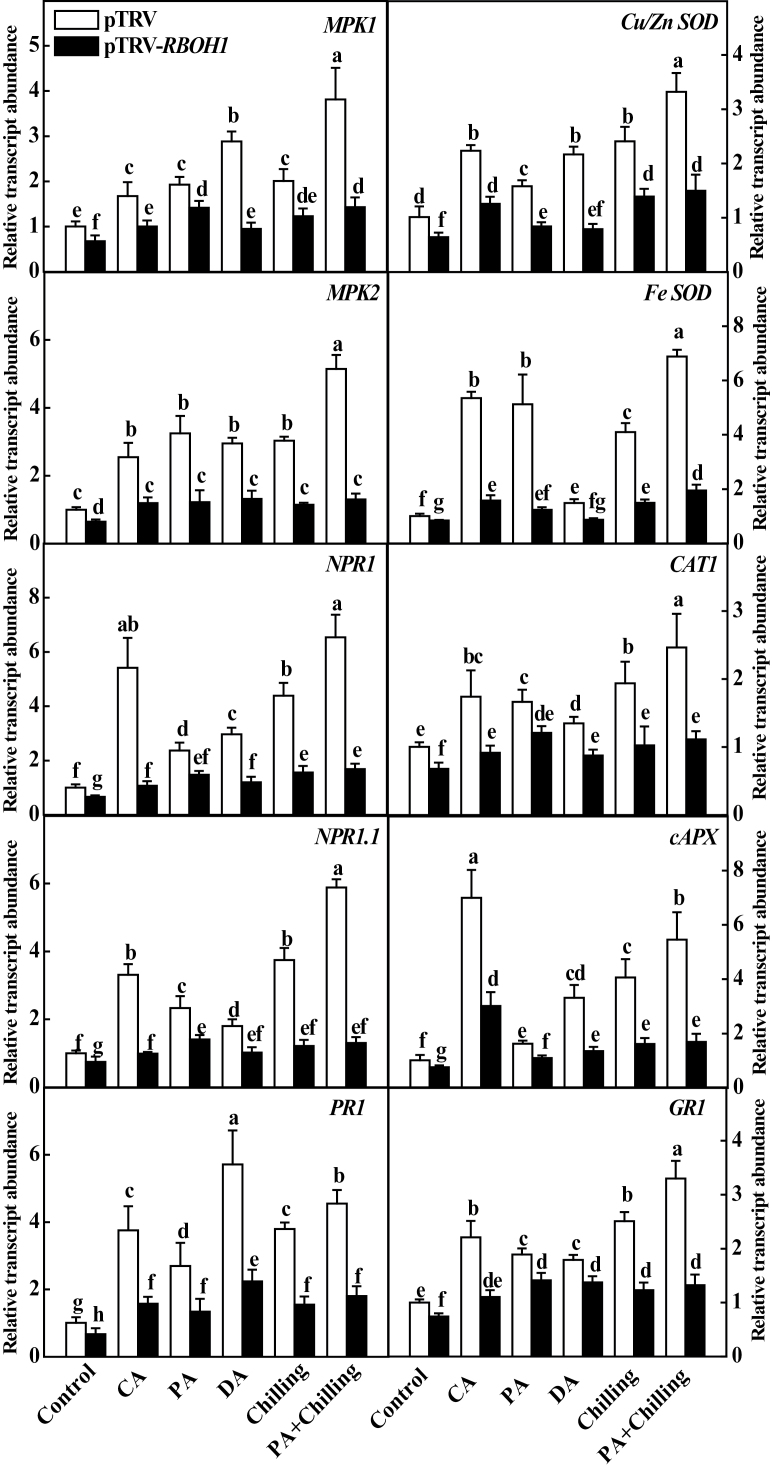
Relative expression of stress-responsive and defence-related genes in response to acclimation and chilling in *RBOH1*-silenced plants. Leaflets in the middle fifth leaf were used. Data are mean±SD of four biological replicates. Different letters above the bars indicate values that are significantly different (*P* < 0.05) according to Tukey’s test. Control, no acclimation; CA, cold acclimation; PA, paraquat acclimation; DA, drought acclimation.

To get insight into the mechanism for cross-acclimation-induced H_2_O_2_ accumulation and enhanced stress tolerance, this study examined changes in the transcript levels of 10 stress-responsive and defence-related genes in VIGS plants in response to different acclimation and chilling stresses (Fig. 5). These genes analysed are *MPK1*, *MPK2*, *NPR1*, *NPR1.1*, *PR1, Fe-SOD*, *Cu/Zn-SOD*, *cAPX*, *CAT1*, and *GR1*. Silencing of *RBOH1* resulted in significant decreases in the transcript levels of all these genes (Fig. 5). In contrast, all acclimation treatments induced increased transcript levels of these genes, ranging from 2- to 8-fold in pTRV plants. Importantly, silencing of *RBOH1* compromised CA-, PA-, DA-, and chilling-induced upregulation of all 10 genes (Fig. 5). Similarly, chilling also induced transcription of these stress-responsive and defence-related genes, and the increases were more significant in PA plants (Fig. 5). However, PA- and chilling-induced transcription was again compromised in pTRV*-RBOH1* plants.

**Fig. 6.**
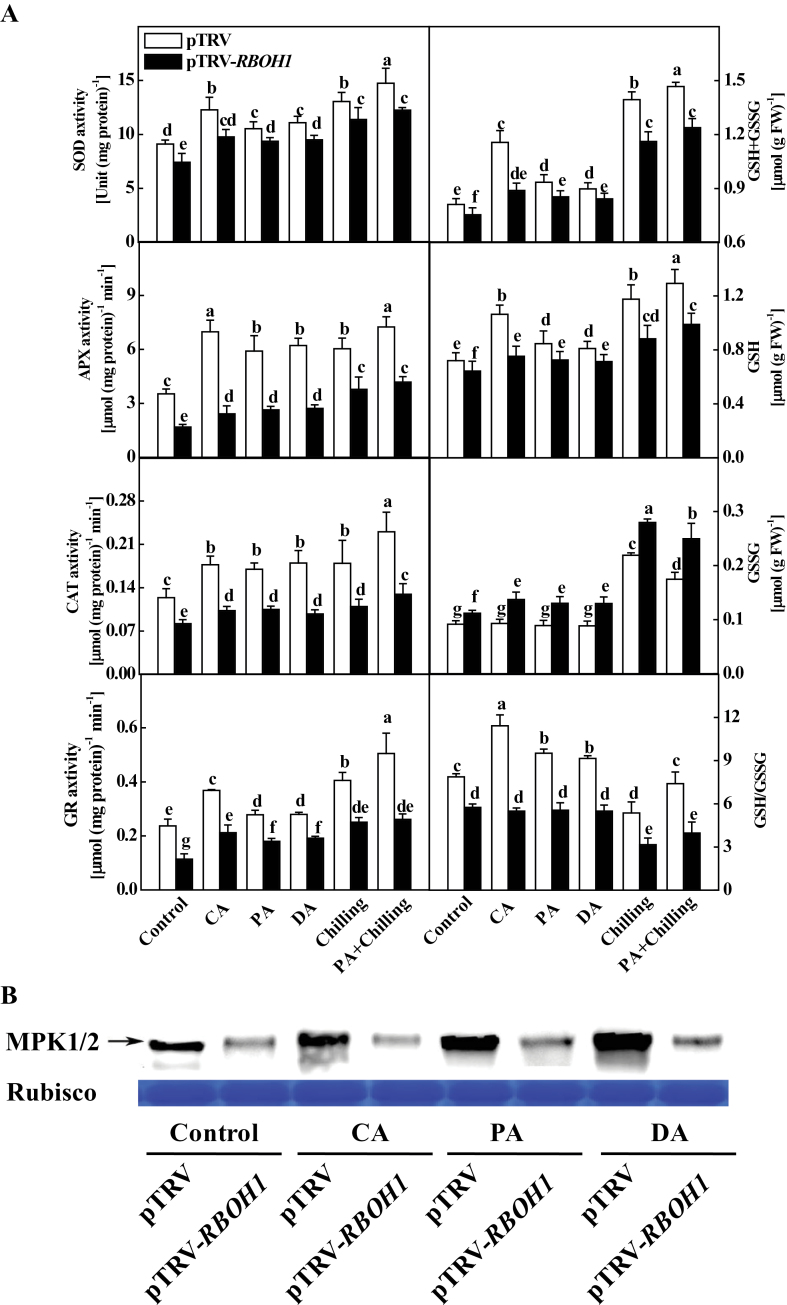
Changes in antioxidant enzyme activities, glutathione homeostasis, and MPK1/2 activation in response to acclimation and chilling in *RBOH1*-silenced plants. (A) Changes in antioxidant enzyme activities and glutathione homeostasis: leaflets in the middle fifth leaf were used; data are mean±SD of four biological replicates; different letters above the bars indicate values that are significantly different (*P* < 0.05) according to Tukey’s test. (B) Changes in MPK1/2 activation: control, no acclimation; CA, cold acclimation; PA, paraquat acclimation; DA, drought acclimation; FW, fresh weight.

This study then analysed changes in the activities of antioxidant enzymes (SOD, APX, CAT, and GR) and glutathione levels (GSH and GSSG) in pTRV and pTRV-*RBOH1* plants after different acclimation and chilling treatments. Similarly to the changes in their transcript levels, silencing of *RBOH1* resulted in significant decreases in the activities of all these antioxidant enzymes (Fig. 6A). On the other hand, silencing of *RBOH1* resulted in significant decreases in GSH content and increased GSSG content, leading to a substantial reduction in both overall GSH+GSSG content and GSH/GSSG ratio (Fig. 6A). In contrast, chilling induced increased GSH and GSSG accumulation and decreased GSH/GSSG ratios. Interestingly, CA, PA, and DA all significantly induced GSH accumulation and reduced GSSG content in pTRV plants, causing sharp increases in total GSH+GSSG and GSH/GSSG ratio (Fig. 6A). Importantly, silencing of *RBOH1* abolished acclimation- and chill-induced increases in GSH content but busted GSSG accumulation, leading to significant decreases in GSH/GSSG ratios (Fig. 6A).

In agreement with the increase in transcript levels of *MPK1* and *MPK2*, MPK1/2 activation was also induced in pTRV plants after CA, PA, and DA. Silencing of *RBOH1* led to a 70% reduction in MPK1/2 activation when compared to that of pTRV plants. Significantly, CA, PA, and DA treatments all failed to induce MPK1/2 activation in pTRV-*RBOH1* plants (Fig. 6B), suggesting that H_2_O_2_ at the apoplast is essential for activation of MPK1/2.

### Role of MAPK1 and 2 activation in acclimation-induced cross-tolerance

**Fig. 7.**
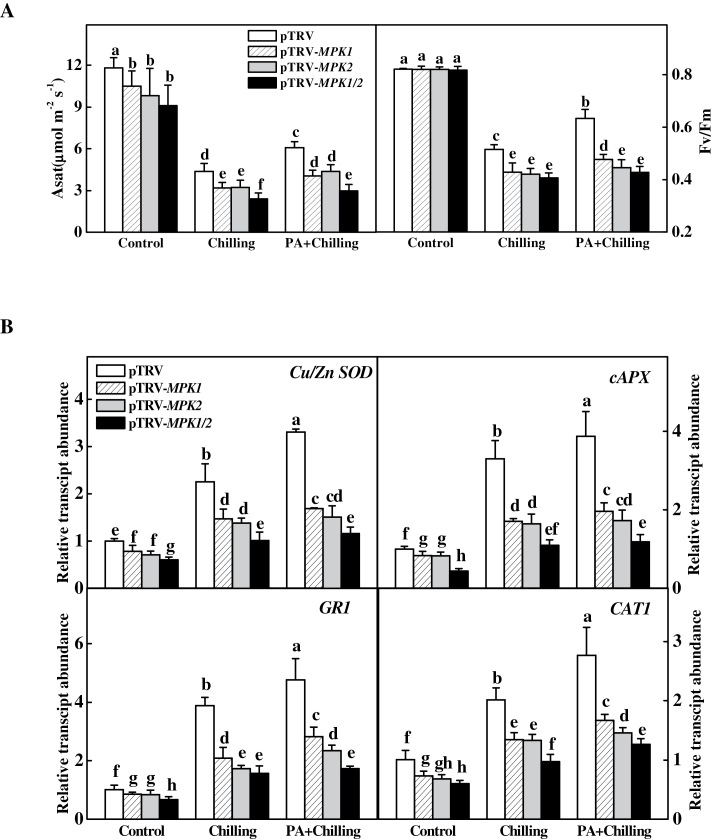
Changes in light-saturated CO_2_ assimilation rate (Asat) and maximum quantum yield of PSII (*F*_v_/*F*_m_) (A) and expression of antioxidant-related genes (B) in response to chilling stress after paraquat acclimation in *MPK1, MPK2-*silenced, and *MPK1/2* co-silenced plants. Leaflets in the middle fifth leaf were used. Data are mean±SD of four biological replicates. Different letters above the bars indicate values that are significantly different (*P* < 0.05) according to Tukey’s test. Control, no acclimation; PA, paraquat acclimation.

To determine the role of MPK1 and 2 in H_2_O_2_-mediated cross-tolerance, this study compared PQ-induced tolerance in plants silenced with *MPK1* (pTRV-*MPK1*), *MPK2* (pTRV-*MPK2*), and *MPK1/2* co-silenced (pTRV-*MPK1/2*) plants with that of pTRV plants for their differences in Asat and *F*_v_/*F*_m_ after chilling at 4 °C. pTRV-*MPK1*, pTRV-*MPK2*, and pTRV-*MPK1/2* plants grew weaker than pTRV plants, and Asat values of those plants were also lower than that of pTRV plants, but *F*_v_/*F*_m_ values were similar to that of pTRV plants when they were grown under the normal environment (Fig. 7A, Supplementary S1B). However, pTRV-*MPK1*, pTRV-*MPK2*, and pTRV-*MPK1/2* plants showed lower Asat and *F*_v_/*F*_m_ values than pTRV plants after exposure to a chilling at 4 °C for 3 d. Importantly, silencing of *MPK1*, *MPK2*, and *MPK1/2* all compromised PA-induced chilling tolerance, as indicated by decreases in both Asat and *F*_v_/*F*_m_.

This study then examined the transcript levels of antioxidant-related genes in PQ-acclimated pTRV-*MPK1*, pTRV-*MPK2*, and pTRV-*MPK1/2* plants after chilling. Silencing of *MPK1* and *MPK2*, or *MPK1*/*2* all resulted in decreased transcript levels of *Cu/Zn-SOD*, *cAPX*, *GR1*, and *CAT1* in the normal environment, and blocked CA- and PA-induced increases in the transcript levels (Fig. 7B). Furthermore, cosilencing of *MPK1* and *2* resulted in substantially lower transcript levels of these genes than silencing of either *MPK1* or *MPK2* (Fig. 7B). All these results suggested that MPK1 and MPK2 are necessary components for acclimation-induced stress tolerance.

## Discussion

There have been many reports about acclimation-induced stress tolerance in plants. For example, wounding acclimation enhanced salt tolerance in tomato while heat and salt acclimation increased stress response against anoxia in *Arabidopsis* and UV in barley (Capiati *et al.*, 2006; Banti *et al.*, 2008; Carkirlar *et al.*, 2008). The current study found that cold acclimation not only increased tolerance to chilling but also to drought and photooxidative stress, and it is also true for other acclimation, suggesting that pretreatment of different types of mild abiotic stresses could induce a common effect on tolerance to a spectrum of stresses.

This work found that *RBOH1*-dependent H_2_O_2_ production plays a critical role in acclimation-induced stress tolerance in tomato plants. ROS, especially H_2_O_2_, produced at the apoplast play an indispensable role in signal recognition and transduction in plant growth, development, and stress response. Hormones such as ABA and BRs could trigger apoplastic H_2_O_2_ generation while exogenous H_2_O_2_ significantly increases tolerance against chilling, paraquat, and high light in plants such as potato and cucumber (Wu *et al.*, 1995; Kwak *et al.*, 2003; Xia *et al.*, 2009). Meanwhile, cold-acclimation-induced cold tolerance is associated with increased H_2_O_2_ accumulation, and loss of function of RBOHs resulted in reduced tolerance against biotic and abiotic stresses (Dat *et al.*, 2003; Zhou *et al.*, 2012). The current study found that not only CA but also DA and PA induced *RBOH1* transcription, NADPH oxidase activity, and H_2_O_2_ accumulation at the apoplast. In tomato, *RBOH1* is involved in responsiveness to wounding (Sagi *et al.*, 2004). The current study found that silencing of *RBOH1* compromised acclimation-induced tolerance, transcription of stress-responsive and defence-related genes, and activation of MPK1/2. Taken together, these findings indicate that H_2_O_2_ is a universal signalling molecule in acclimation and that *RBOH1*-dependent H_2_O_2_ generation plays a critical role in acclimation-induced cross-tolerance in tomato plants.

The responses of plants to acclimation and stress are frequently associated with changes in the cellular redox state (Foyer and Noctor, 2005). The current study found that acclimation leads to increases in both the activity of antioxidant enzymes and GSH accumulation and, ultimately, to an increase in the GSH/GSSG ratio while silencing of *RBOH1* abolished induction of such changes (Fig. 6). H_2_O_2_ could influence the expression of stress-responsive and defence-related genes and the activity of antioxidant enzymes, GSH biosynthesis, and defence responses in plants (Desikan *et al.*, 2001; Vandenabeele *et al.*, 2003; Xia *et al.*, 2009). In agreement with these studies, these acclimations induced significant changes in the antioxidant-related gene transcript levels, enzyme activities, and redox homeostasis, and this effect was highly dependent on accumulation of *RBOH1*-induced H_2_O_2_ after acclimation in this study (Figs. 5 and 6). Importantly, acclimated plants exhibited higher GSH/GSSG ratios than those of unacclimated plants during acclimation and chilling. Glutathione homeostasis could influence plant metabolism and stress response by modifying the activity of redox-sensitive enzymes such as Rubisco activase through reduction/oxidation of disulphide bridges/sulphydryl groups and glutathionylation of sulphydryl groups (Szalai *et al.*, 2009; Jiang *et al.*, 2012). Accordingly, acclimation-induced changes in glutathione homeostasis may partially contribute to increased CO_2_ assimilation in acclimated plants (Fig. 6A). There is also evidence that the glutathione redox state is involved in the regulation of the transcription and stability of defence-related genes and proteins (Baena-Gonzalez and Aro, 2002; Mou *et al.*, 2003). Interestingly, there were significant increases in the transcript levels of *NPR1* and *PR1*, which are essential regulators for the onset of systemic acquired resistance in acclimated plants, and the increases were again abolished in *RBOH1*-silenced plants (Fig. 5). Recently, this study group reported that BRs enhance tolerance against chilling/PQ stresses and CO_2_ assimilation by H_2_O_2_-dependent change of the glutathione redox state (Xia *et al.*, 2009; Jiang *et al.*, 2012). Therefore, acclimation-induced stress tolerance appears to involve a conserved stress-responsive and defence-related mechanism that is activated by the RBOHs-H_2_O_2_-GSH/GSSG-dependent signalling pathway. It will be of great interest to study whether these acclimations could induce resistance against biotic stress.

MAPK cascades are known to mediate the transduction of environmental and developmental signals into intracellular responses (Mizoguchi *et al.*, 1996; Teige *et al.*, 2004). Tomato *MPK1/2* are orthologues of *Arabidopsis MPK6*, which is involved in plant responses to pathogens (Menke *et al.*, 2004; Schikora *et al.*, 2011). Several studies revealed that *MPK1/2* function in host-specific Avr Pto-dependent resistance to the bacterial pathogen *P. syringae* (Ekengren *et al.*, 2003; Pedley and Martin, 2004) and in *Mi-1*-mediated resistance to aphids and herbivorous insects in tomato plants (Li *et al.*, 2006; Kandoth *et al.*, 2007). The current study observed that pTRV-*MPK1*, pTRV-*MPK2*, and pTRV-*MPK1/2* plants showed decreased tolerance against chilling, extending an earlier observation that *SlMPK1* and *SlMPK2* are not only involved in plant pest resistance but also in plant tolerance to abiotic stresses (Nie *et al.*, 2013). Significantly, all acclimations increased MPK1/2 activation while silencing of *MPK1* and *MPK2* abolished PA-induced transcription in antioxidant genes and chilling tolerance, as observed in BR-induced stress response and MPK1/2 activation (Fig. 7; Nie *et al.*, 2013). All these results indicate that *MPK1/2* play an important role in acclimation-induced stress response.

Studies have revealed that there is an interesting relationship between NADPH oxidase-produced ROS and MAPK activation in plants exposed to various stresses or stimuli (Yoshioka *et al.*, 2003; Pitzschke and Hirt, 2006; Nie *et al.*, 2013). Several plant hormones such as ABA and BRs as well as ABA- and BR-induced H_2_O_2_ are known to activate MAPKs, which are involved in ABA- and BR-induced antioxidant defence responses (Zhang *et al.*, 2006; Lin *et al.*, 2009). There are also evidences that MAPKs are involved in regulation of RBOHs in plants (Yoshioka *et al.*, 2003; Asai *et al.*, 2008; Zhang *et al.*, 2010). The current study found that acclimation induced H_2_O_2_ accumulation and activation of MPK1/2, while silencing of *RBOH1* resulted in reduced MPK1/2 activation and abolished acclimation-induced activation of MPK1/2, suggesting that NADPH oxidase-produced H_2_O_2_ regulated MPK1/2 activation during acclimation (Fig. 6B). Very recently, (Nie *et al.*, 2013) found that there exists a positive feedback circuit between *RBOH1* and *MPK1/2* in BRs signalling cascade. It is, therefore, plausible that H_2_O_2_-activated MAPKs are also important in maintaining H_2_O_2_ generation by NADPH oxidase during acclimation.

Cross-tolerance could be generated by signalling cross-talk by means of shared components that interrelate the signalling cascades triggered by each type of stress, and, as a result, one type of stress can activate responses that lead to tolerance to other types of stresses (Capiati *et al.*, 2006). It is not surprising that other signalling molecules such as NO and Ca^2+^ could also be involved in acclimation and cross-tolerance. In tomato, CDPK1, a Ca^2+^-dependent protein kinase, participates in responses to wounding and salt stress (Capiati *et al.*, 2006). More recently, it has been found that nitrate reductase-dependent NO generation participates in cold-induced chilling tolerance (Zhao *et al.*, 2009). ROS such as H_2_O_2_ are also involved in regulation of the generation or the levels of these signals, which may contribute to the critical role of ROS in acclimation and subsequent cross-tolerance.

In conclusion, this work demonstrated that mild cold, PQ, or drought pretreatment can result in enhanced tolerance to multiple abiotic stresses by triggering a significant increase in endogenous H_2_O_2_ level at the apoplast, which is associated with upregulation of *RBOH1* transcription. The elevated H_2_O_2_ induced transcription of a subset of stress- and defence-related genes, activity of antioxidant enzymes, reduced cellular redox status, and activation of MPK1/2. Silencing of *RBOH1* and *MPK1/2* both compromised acclimation-induced stress tolerance and associated changes in gene transcription. All these findings support the involvement of a *RBOH1*-dependent activation of MPK1/2 in acclimation-induced cross-tolerance in plants.

## Supplementary material

Supplementary data are available at *JXB* online.

Supplementary Fig. S1. Relative mRNA abundance of *MPK1* and *MPK2* and phenotypes in VIGS plants.

Supplementary Fig. S2. Cross-acclimation-induced ROS accumulation in pTRV and pTRV-*RBOH1* plants.

Supplementary Table S1. Primers for qRT-PCR.

Supplementary Data
