# Supplementary material for: RBOH1-dependent H2O2 production and subsequent activation of MPK1/2 play an important role in acclimation-induced cross-tolerance in tomato
Source: J Exp Bot. 2013 Dec 9;65(2):595–607. doi: 10.1093/jxb/ert404 (PMC3904713; doi:10.1093/jxb/ert404)
Supplement: Supplementary Data [file supp_65_2_595__index.html]

 RBOH1-dependent H2O2 production and subsequent activation of MPK1/2 play an important role in acclimation-induced cross-tolerance in tomato — RBOH1-dependent H2O2 production and subsequent activation of MPK1/2 play an important role in acclimation-induced cross-tolerance in tomato — Supplementary Data 

# *RBOH1*-dependent H2O2 production and subsequent activation of MPK1/2 play an important role in acclimation-induced cross-tolerance in tomato

## Supplementary Data

Data files

**Files in this Data Supplement:**

- Supplementary Data - Supplementary Data
