# Supplementary material for: RBOH1-dependent H2O2 production and subsequent activation of MPK1/2 play an important role in acclimation-induced cross-tolerance in tomato
Source: J Exp Bot. 2013 Dec 9;65(2):595–607. doi: 10.1093/jxb/ert404 (PMC3904713; doi:10.1093/jxb/ert404)
Supplement: Supplementary Data [file supp_ert404_jexbot111062_file001.pdf]

Journal of Experimental Botany

RBOH1-dependent H_2O_2 production and subsequent activation of MPK1/2 play an important role in acclimation-induced cross tolerance in tomato.

J Q Yu, Yanhong Zhou, Jie Zhou, Xiaojian Xia, Kai Shi, and Zhixiang Chen.

SUPPLEMENTARY DATA

Supplementary Figure S1

A

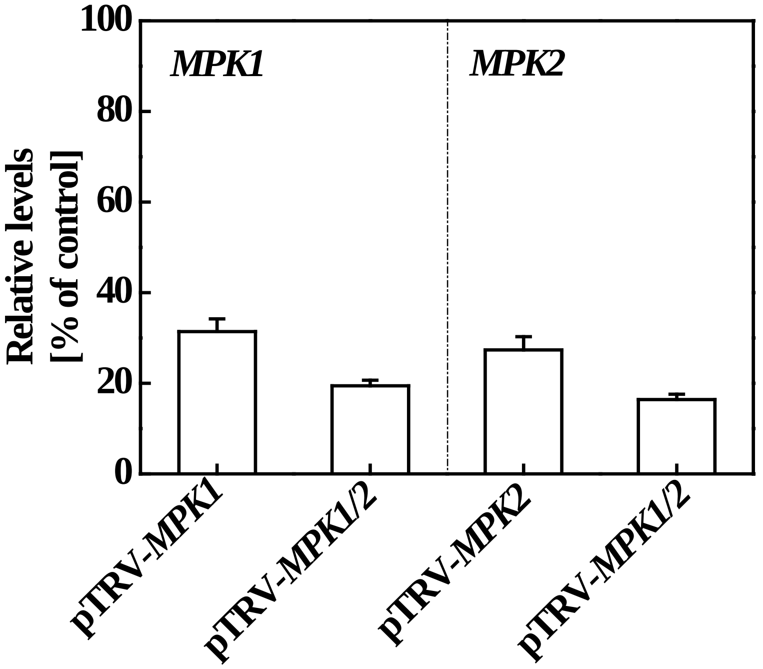

B

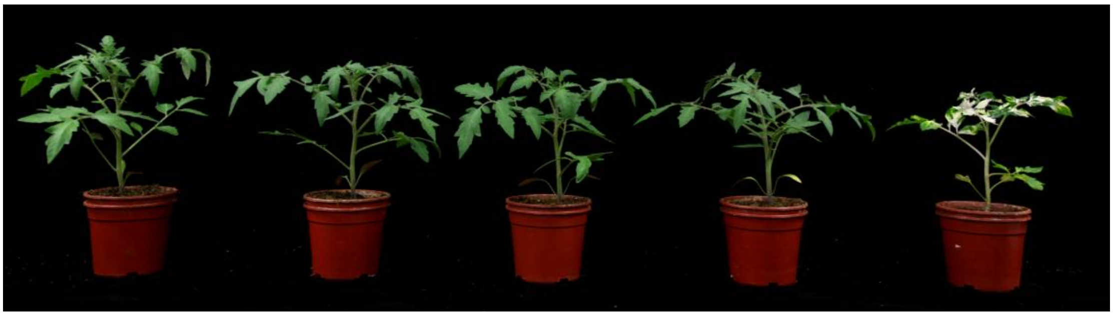

pTRV **pTRV-*MPK1*** **pTRV-*MPK2*** **pTRV-*MPK1/2*** **pTRV-*PDS***

Figure S1. Relative mRNA abundance of *MPK1 and MPK2* in VIGS plants (A) and the phenotypes of VIGS plants (B). The expression of *MPK1* and *MPK2* were analyzed at 30 d after *Agrobacterium*-infection. Data are the means of the 5th leaf of six silenced plants (±SD). The levels were expressed as percentages of the mean levels in control pTRV plants which were defined as 100%. The bleaching of the pTRV-*PDS* (phytoene desaturase) plants was used to monitor the progress of gene silencing.

Supplementary Figure S2.

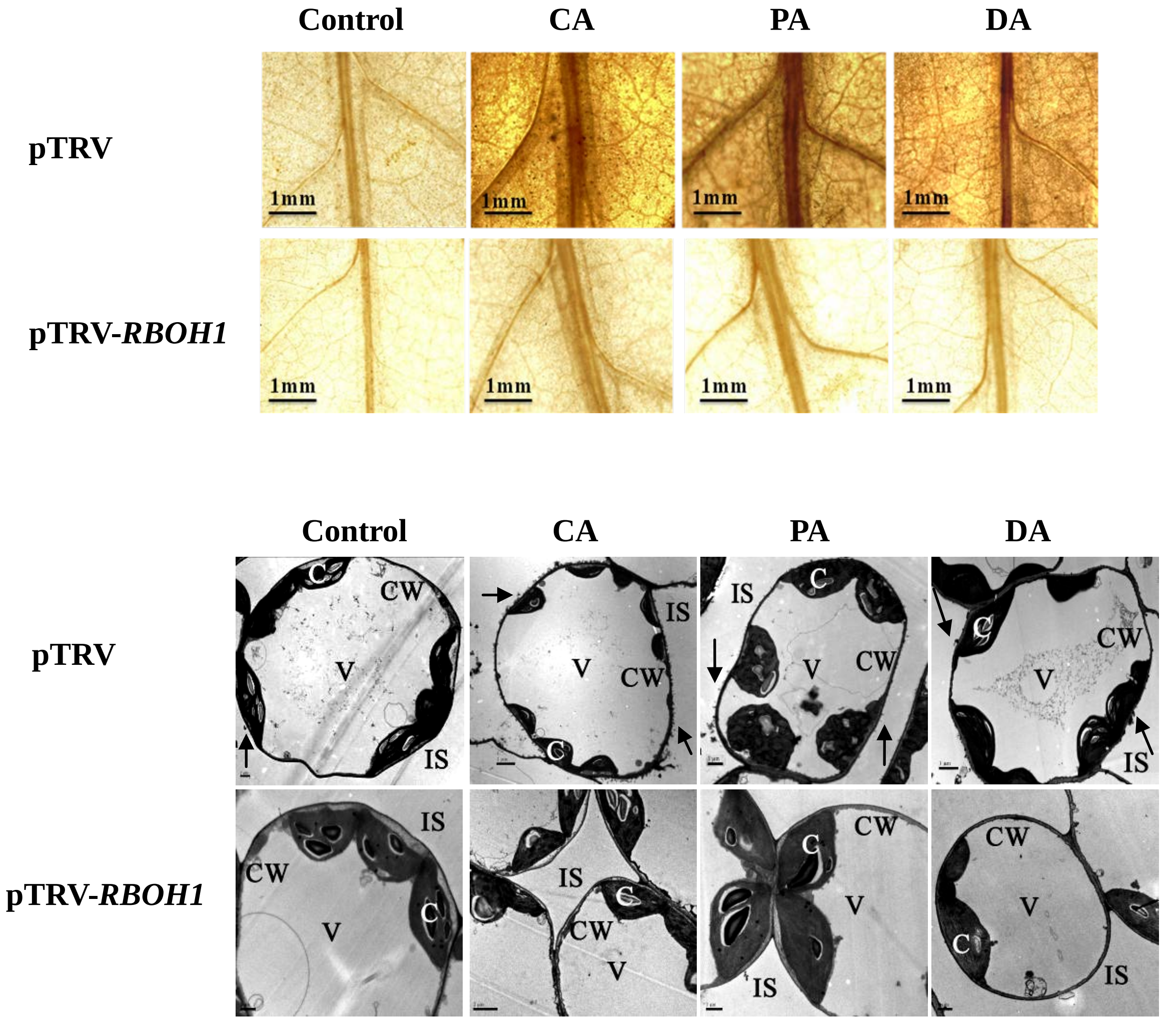

Figure S2. Cross-acclimation induced ROS accumulation in pTRV and pTRV-*RBOH1* plants

A, The *in situ* detection of H_2O_2 in leaves. The leaf segments were loaded with DAB and incubated for 6 h. The H_2O_2 accumulation was detected by microscope. Bar = 1.0 mm.

B, The cytochemical localization of H_2O_2 accumulated in the mesophyll cells of leaves detected with $CeCl_3$ staining and transmission electron microscopy. Arrows, $CeCl_3$ precipitates; C, chloroplast; CW, cell wall; V, vacuole; IS, intercellular space.

Control, non acclimation; CA, cold acclimation; PA, paraquat acclimation; DA, drought acclimation.

Supplementary Table S1. Primers for qRT-PCR.

Gene	Accession Numbers	Forward primer	Reverse primer
RBOH1	Sl08g081690	5’-GGAGCTCCAGCACAAGATTA-3’	5’-CTTGTTGCAGCACTCATGTC-3’
MPK1	Sl12g019460	5’- GCTGACAGATTGTTGCAGGT -3’	5’- TCCACCCCATAAAGATACATCA -3’
MPK2	Sl08g014420	5’- TACTCGCTCGTTTGCTGTTG -3’	5’- TTGGAGTACAGGAAAACAATGG -3’
NPR1	Sl07g040690	5’- GATAGCAACACGAAGCTGGA -3’	5’- GGCCTACAAGCTACATGCAA -3’
NPR1.1	Sl07g044980	5’- TGGACATACAGCCCTATCCA -3’	5’- AGCCGATCTTGGAGTCTTT -3’
PR1	Sl01g106600	5’- CGTGCAGGCAGTTAATTTGT -3’	5’- CACAAACCACCATTCACCAT -3’
Fe-SOD	Sl06g048410	5’- TAAATAGAGACTTTGGTTCC -3’	5’- TATATTTGCCTCTTAACCCT -3’
Cu/Zn-SOD	Sl11g066390	5’- GGCCAATCTTTGACCCTTTA -3’	5’- AGTCCAGGAGCAAGTCCAGT -3’
cAPX	Sl06g005160	5’- TCTGAATTGGGATTTGCTGA -3’	5’- CGTCTAACGTAGCTGCCAAA -3’
GR1	Sl09g091840	5’- TTGGTGGAACGTGTGTTCTT -3’	5’- TCTCATTCACTTCCCATCCA -3’
CAT1	Sl12g094620	5’- TGATCGCGAGAAGATACCTG -3’	5’-CTTCCACGTTCATGGACAAC-3’
Actin	Sl11g005330	5’- TGTCCCTATTTACGAGGGTTATGC -3’	5’- CAGTTAAATCACGACCAGCAAGAT -3’
